# HTLV-1 intragenic viral enhancer influences immortalization phenotype *in vitro*, but is dispensable for persistence and disease development in animal models

**DOI:** 10.3389/fimmu.2022.954077

**Published:** 2022-07-25

**Authors:** Victoria Maksimova, Susan Smith, Jaideep Seth, Cameron Phelps, Stefan Niewiesk, Yorifumi Satou, Patrick L. Green, Amanda R. Panfil

**Affiliations:** ^1^ Department of Veterinary Biosciences, Center for Retrovirus Research, The Ohio State University, Columbus, OH, United States; ^2^ Division of Genomics and Transcriptomics, Joint Research Center for Human Retrovirus Infection, Kumamoto University, Kumamoto, Japan; ^3^ Comprehensive Cancer Center and Solove Research Institute, The Ohio State University, Columbus, OH, United States

**Keywords:** retrovirus, HTLV-1, enhancer, immortalization, persistence, transcription

## Abstract

Human T-cell leukemia virus type 1 (HTLV-1) is the causative infectious agent of adult T-cell leukemia/lymphoma (ATL) and chronic neurological disease. The disparity between silenced sense transcription versus constitutively active antisense (Hbz) transcription from the integrated provirus is not fully understood. The presence of an internal viral enhancer has recently been discovered in the Tax gene near the 3’ long terminal repeat (LTR) of HTLV-1. *In vitro*, this enhancer has been shown to bind SRF and ELK-1 host transcription factors, maintain chromatin openness and viral gene transcription, and induce aberrant host gene transcription near viral integration sites. However, the function of the viral enhancer in the context of early HTLV-1 infection events remains unknown. In this study, we generated a mutant Enhancer virus (mEnhancer) and evaluated its effects on HTLV-1-mediated *in vitro* immortalization, establishment of persistent infection with an *in vivo* rabbit model, and disease development in a humanized immune system (HIS) mouse model. The mEnhancer virus was able to establish persistent infection in rabbits, and there were no significant differences in proviral load or HTLV-1-specific antibody responses over a 25-week study. However, rabbits infected with the mEnhancer virus had significantly decreased sense and antisense viral gene expression at 12-weeks post-infection. HIS mice infected with wt or mEnhancer virus showed similar disease progression, proviral load, and viral gene expression. While mEnhancer virus was able to sufficiently immortalize primary T-lymphocytes in cell culture, the immortalized cells had an altered phenotype (CD8^+^ T-cells), decreased proviral load, decreased sense and anti-sense gene expression, and altered cell cycle progression compared to HTLV-1.wt immortalized cells (CD4^+^ T-cells). These results suggest that the HTLV-1 enhancer element alone does not determine persistence or disease development but plays a pivotal role in regulating viral gene expression.

## Introduction

Human T-cell leukemia virus type 1 (HTLV-1) is a retrovirus with an estimated 5-10 million individuals infected worldwide including endemic regions in Southwestern Japan, Sub-Saharan Africa, South America, and the Caribbean (1). HTLV-1 infection can lead to the development of an aggressive CD4^+^ T-cell malignancy, adult T-cell leukemia/lymphoma (ATL) (2–4), or a neurological disorder called HTLV-1-associated myelopathy/tropical spastic paraparesis (HAM/TSP) (5, 6). An approximate 5-10% of persons infected with HTLV-1 develop ATL or HAM/TSP and generally undergo a decades-long clinical latency period before the onset of disease symptoms (7). The factors which control the timing and the disease course in persons infected with HTLV-1 remain to be fully elucidated.

The regulation of viral gene expression from the integrated provirus is key to HTLV-1 persistence and the pathogenic outcomes of infection. Tax, the initial dominant transcript produced from the provirus, amplifies viral sense strand transcription and drives cellular transformation through interactions within major signaling pathways, including NF-κB and AP-1 (8). HTLV-1 also encodes a gene on the antisense genome strand of the provirus, Hbz, which functions to maintain the proliferation and survival of leukemic cells (9–11). While immune surveillance directs the selection of cells in which Tax expression is lost or transient (12–15), transcription of Hbz is constitutive (16, 17). It has been demonstrated that Hbz is critical for establishment of efficient viral persistence in the New Zealand White (NZW) rabbit model of HTLV-1 infection (18). It has also been shown that both Hbz mRNA and protein support T-cell proliferation (9, 10); however, the mechanisms governing the balance between sense and antisense HTLV-1 transcription require further investigation.

Recently, an intragenic viral enhancer was discovered within the HTLV-1 provirus through a screen for transcriptional regulatory regions using proviral DNA-capture sequencing (19). The transcription factors SRF and ELK-1 were found to bind to this region and play a role in enhancer activity. It was demonstrated *in vitro* that the HTLV-1 enhancer maintains chromatin openness, regulates viral gene transcription, and induces aberrant host gene transcription near viral integration sites. Given its potential contributions to frequent transcription driven from the 3’ LTR and constitutive Hbz expression, we aimed to characterize the role of the viral enhancer during early HTLV-1 infection events *in vitro* and *in vivo*. Our results showed that the viral enhancer is dispensable for persistence using a rabbit model of infection. However, disruption of the viral enhancer did significantly decrease both sense and antisense viral gene expression during infection. Using a HIS mouse disease model, loss of the viral enhancer did not affect HTLV-1-mediated disease progression. Absence of the viral enhancer did not affect immortalization efficiency in cell culture but did alter the immortalized cell phenotype to a predominance of CD8^+^ T-cells. Also, HTLV-1-immortalized T-cells lacking the viral enhancer had decreased proviral load, decreased sense and anti-sense gene expression, lower levels of cellular apoptosis, and altered cell cycle progression.

## Materials and methods

### Cell culture

Human embryonic kidney (HEK) 293T cells were cultured in Dulbecco’s modified Eagle’s medium (DMEM) (Gibco, Thermo Fisher Scientific, Waltham, MA) supplemented with 10% fetal bovine serum (FBS), 100 U/mL penicillin, 100 μg/mL streptomycin, and 2 mM L-glutamine. Jurkat cells, an HTLV-1-negative, transformed human T-cell line, were cultured in Roswell Park Memorial Institute (RPMI) 1640 medium (Gibco, Thermo Fisher Scientific, Waltham, MA) supplemented with 10% FBS, 100 U/mL penicillin, 100 μg/mL streptomycin, and 2 mM L-glutamine. Parental human 729.B control cells (lymphoblastoid B-cell line) and 729 HTLV-1 producer cell lines were cultured in Iscove’s DMEM (Mediatech, Inc. Manassas, VA) supplemented with 10% FBS, 100 U/mL penicillin, 100 μg/mL streptomycin, and 2 mM L-glutamine. Human PBMCs (hPBMCs) were isolated from whole blood freshly collected from healthy donors using Ficoll-Paque PLUS (Cytiva, Marlborough, MA). Protocols for blood collection from human donors were approved by the Ohio State University Institutional Review Board. hPBMCs and early passage, HTLV-1-immortalized primary human T-cell lines (PBL) were cultured in RPMI 1640 supplemented with 20% FBS, 100 U/mL penicillin, 100 μg/mL streptomycin, 2 mM L-glutamine, and 10 U/mL recombinant human interleukin-2 (hIL-2; Roche Diagnostics GmbH, Mannheim, Germany). All cells were maintained in a humidified atmosphere of 5% CO_2_ and air at 37°C.

### Plasmids and cloning

**Figure 1:**
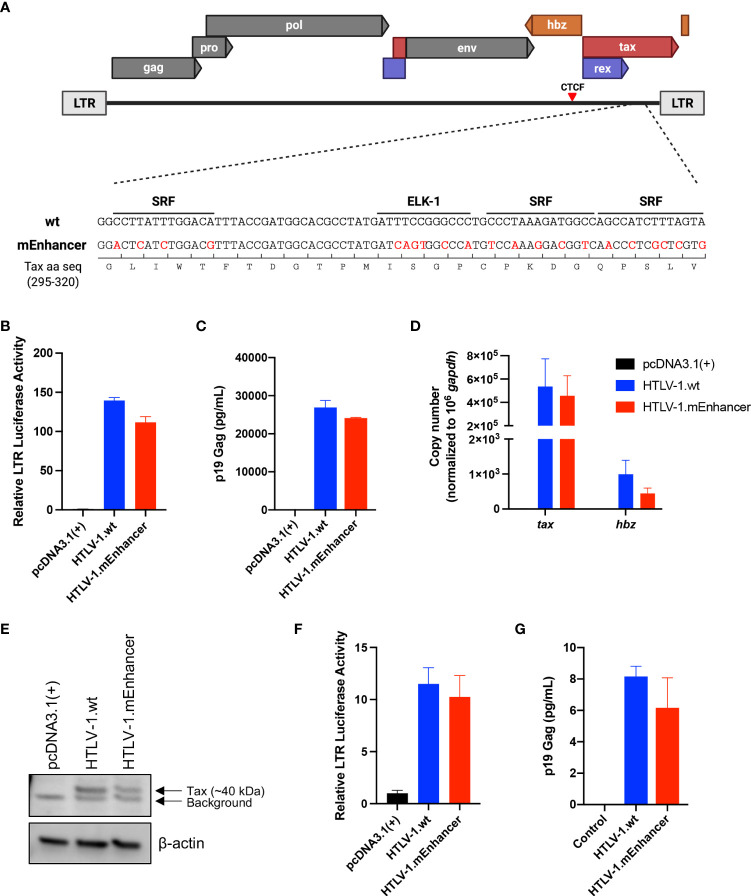
Generation and characterization of HTLV-1.mEnhancer proviral clone *in vitro*. The mEnhancer region was cloned into the HTLV-1 proviral plasmid ACHneo by overlap extension PCR. **(A)** Alignment of the mEnhancer region and the wild-type region from the HTLV-1 molecular clone ACHneo. Mutations span the consensus binding sequences for the transcription factors SRF and ELK-1. **(B)** HEK293T cells were co-transfected with the pcDNA3.1(+) empty vector, HTLV-1.wt, or mutant (HTLV-1.mEnhancer) proviral plasmid, an HTLV-1 LTR-firefly luciferase construct, and a TK-Renilla luciferase construct. 72h post-transfection, cells and supernatant were collected for dual luciferase assay and ELISA to detect HTLV-1 p19 Gag **(C)**, respectively. Relative LTR luciferase activity was determined by normalizing firefly luciferase relative light units to Renilla, and the empty vector control was set as 1. Each condition was performed in duplicate. **(D)** RNA was extracted from transfected cells for cDNA synthesis, followed by qPCR to detect *tax* and *hbz* mRNA levels. Copy number is shown normalized to 1 x 10^6^ *gapdh* copies. **(E)** Cells pellets from transfected cells were lysed and total protein was quantified by BCA assay. Equivalent amounts of protein were subjected to SDS-PAGE and immunoblotting to detect Tax expression. β-actin was used as a loading control. Arrows are used to distinguish bands representative of Tax protein expression from background. **(F)** The HTLV-1-negative T-cell line, Jurkat, was co-transfected with the pcDNA3.1(+) empty vector, HTLV-1.wt, or mutant (HTLV-1.mEnhancer) proviral plasmid, an HTLV-1 LTR-firefly luciferase construct, and a TK-Renilla luciferase construct. 72h post-transfection, cells and supernatant were collected for dual luciferase assay and ELISA to detect HTLV-1 p19 Gag **(G)**, respectively. Relative LTR luciferase activity was determined as described above. Each condition was performed in duplicate. Graphs represent data generated from duplicate samples and error bars represent standard deviation (SD). Statistical significance was determined by unpaired t test.

The infectious HTLV-1 proviral clone ACH (HTLV-1.wt) has been previously characterized (20, 21). The mutant Enhancer (mEnhancer) region (19) was created in ACH through subcloning and overlap extension PCR (22) using annealed oligos containing mutations in the SRF/ELK-1 binding sites (nucleotide mutations shown in **Figure 1A**). The nucleotide mutations do not result in changes to the overlapping Tax amino acid sequence. Proviral plasmid DNA was isolated and purified using cesium chloride density gradient centrifugation. The HTLV-1 5’LTR-luciferase reporter plasmid (LTR-1-Luc) and thymidine kinase (TK)-Renilla transfection efficiency control plasmid were described previously (23). The pcDNA™3.1^(+)^ negative control (empty vector) was purchased from Invitrogen (Carlsbad, CA).

### Transfections, luciferase reporter assays, and p19 Gag ELISA

HEK293T cells were plated in 6-well dishes at a density of 3 x 10^5^ cells in 2 mL media. Cells were co-transfected with 880 ng empty vector or proviral plasmid DNA, 100 ng LTR-1-Luc, and 20 ng TK-Renilla using Lipofectamine™ 2000 Transfection Reagent (Invitrogen, Carlsbad, CA) at a 3:1 ratio of reagent (μL) to DNA (μg). Each condition was performed in duplicate. 72h post-transfection, cell supernatants were collected to measure HTLV-1 p19 Gag production using the RETRO-TEK HTLV p19 Antigen ELISA (ZeptoMetrix Corporation, Buffalo, NY). Cells were collected by centrifugation for RNA extraction, RT, and subsequent quantitative PCR to detect Tax and Hbz gene expression (see Materials and Methods: Quantitative PCR). Cells were also collected for luciferase assay or immunoblotting (see Materials and Methods: Immunoblotting). Luciferase assays were performed by lysing cell pellets in Passive Lysis Buffer (Promega, Madison, WI) and following the manufacturer’s protocol for the Dual-Luciferase^®^ Reporter Assay System (Promega, Madison, WI). Firefly and Renilla luciferase relative light units were measured using the FilterMax F5 Multi-Mode Microplate Reader (Molecular Devices, San Jose, CA). Jurkat cells were plated in a 12-well dish at a density of 8 x 10^5^ cells in 1 mL media. Cells were co-transfected with 2 μg total plasmid DNA (1760 ng empty vector or proviral plasmid DNA, 200 ng LTR-1-Luc, and 40 ng TK-Renilla) using Lipofectamine™ 2000 Transfection Reagent (Invitrogen, Carlsbad, CA) at a 2:1 ratio of reagent (μL) to DNA (μg). 72h post-transfection, cells and supernatant were collected for dual luciferase assay and p19 Gag ELISA, respectively, as described above.

### Generation of HTLV-1 producer cells

Stable producer cell lines were generated by nucleofecting 729.B cells with 2 μg HTLV-1.wt or HTLV-1.mEnhancer proviral plasmid DNA (contains neomycin resistance gene) using the Amaxa^®^ Cell Line Nucleofector^®^ Kit V and Nucleofector™ 2b Device (program X-001) according to the manufacturer’s protocol (Lonza Cologne AG, Cologne, Germany). Seventy-two hours post-transfection, cells were placed under 1 mg/mL G418 selection (Gibco, Thermo Fisher Scientific, Waltham, MA). Approximately two weeks after G418 selection, cell supernatant was collected to measure HTLV-1 p19 Gag production by ELISA (ZeptoMetrix Corporation, Buffalo, NY). Polyclonal cells with confirmed p19 production were subjected to single cell dilution. Subsequent single cell clones were plated at a density of 1 x 10^6^ cells in 2 mL media and supernatant was collected after 24h for ELISA to obtain normalized p19 Gag values. 729 HTLV-1.mEnhancer clones with p19 Gag production comparable to established 729 HTLV-1.wt producer cells were used for *in vitro* immortalization assays and *in vivo* inoculation. Genomic DNA was isolated from single cell clones using the DNeasy Blood & Tissue Kit (QIAGEN, Hilden, Germany) and mEnhancer mutations were verified by PCR and Sanger sequencing (see Materials and Methods: Sequencing).

### Immunoblotting

Transfected HEK293T cells and 729 HTLV-1.wt or HTLV-1.mEnhancer producer cell clones were lysed in RIPA buffer with protease inhibitor cocktail (cOmplete™, Mini Protease Inhibitor Cocktail, Roche Diagnostics GmbH, Mannheim, Germany). Total protein was quantitated using the Pierce™ BCA Protein Assay Kit (Thermo Fisher Scientific, Rockford, IL) and FilterMax F5 Multi-Mode Microplate Reader (Molecular Devices, San Jose, CA). Protein was loaded in equal amounts on 4–20% Mini-PROTEAN^®^ TGX™ Precast Protein Gels (Bio-Rad, Hercules, CA) and transferred onto Amersham™ Protran^®^ Western blotting nitrocellulose membranes (Cytiva, Marlborough, MA). Membranes were blocked with 5% milk in PBS with 0.1% Tween-20 and incubated in the following primary antibodies: anti-Tax (rabbit anti-sera), anti-Hbz (rabbit anti-sera), and anti-β-actin (1:5,000; A2228, Sigma-Aldrich, St. Louis, MO). Membranes were incubated in appropriate anti-rabbit or -mouse IgG (H+L) HRP conjugate secondary antibodies (1:5000; W401B or W402B, Promega, Madison, WI). Pierce™ ECL Western Blotting Substrate (Thermo Fisher Scientific, Rockford, IL) was used to develop the membranes and images were captured using an Amersham Imager 600 (GE Healthcare Life Sciences).

### In vitro immortalization assay

Approximately 2 x 10^6^ freshly isolated hPBMCs were co-cultured with 1x 10^6^ lethally irradiated (100 Gy) 729.B control cells, 729 HTLV-1.wt, or 729 HTLV-1.mEnhancer producer cells in 24-well dishes. 10 U/mL hIL-2 was supplied weekly with subsequent media changes. A portion of each irradiated producer cell line was maintained in culture to confirm cell death. Viable cells were enumerated at weekly intervals in triplicate wells for each condition by Trypan Blue exclusion (Gibco, Thermo Fisher Scientific, Waltham, MA). Cell supernatant was collected from enumerated wells for subsequent p19 Gag measurement by ELISA. Two different blood donors were used in our studies. Approximately equivalent numbers of cell lines were generated for both wt and mEnhancer conditions (Donor 1: 11 wt cell lines, 5 mEnhancer cell lines; Donor 2: 4 wt cell lines, 9 mEnhancer cell lines), however with different efficiencies between blood donors. A representative selection of both HTLV-1.wt and HTLV-1.mEnhancer immortalized cell lines from each blood donor was utilized in subsequent experiments.

### Humanized immune system mice

Breeding pairs of NOD.Cg-*Prkd^cscid^ Il2rg^tm1Wjl^*/SzJ mice (NSG strain, specific pathogen free) were purchased from The Jackson Laboratory (Bar Harbor, ME). These mice lack mature T cells, B cells, and functional NK cells, and are deficient in cytokine signaling. Animals were housed in individually ventilated microisolation cages with corncob bedding and provided with commercial pelleted rodent chow and chlorinated reverse-osmosis–purified water without restriction. Cages containing autoclaved bedding were used for cage changes, which were performed in a ventilated biosafety cabinet. Mice were maintained in a room with constant temperature (20 ± 2°C) and relative humidity (50% ± 20%) under a 12:12-h light-dark cycle. The animal use protocol received prior approval by the Institutional Animal Care and Use Committee of The Ohio State University.

Shortly (24 to 48 h) after birth, pups were removed temporarily from the dam and treated with whole-body irradiation at 1 Gy (RS 2000, Rad Source, Suwanee, GA). Each mouse then was injected into the liver with 3 × 10^4^ to 1 × 10^5^ CD34^+^ HUSC (Lonza, Allendale, NJ) in 50 μL PBS (pH 7.4) by using a sterile 26-gauge hypodermic needle. After recovery, pups were returned to their dams, allowed to mature normally, weaned at 21 d, and then housed in groups (maximum, 5 mice per cage). At 10 wk after HUSC engraftment, mice were tested for the presence of human peripheral blood cells. Mice with at least 15% human CD45-positive lymphocytes were infected by intraperitoneal inoculation of 10^7^ lethally irradiated (100 Gy) 729 HTLV-1.wt or 729 HTLV-1.mEnhancer producer cells; an aliquot of cells was maintained in culture to control for irradiation treatment. Animals were euthanized when they lost more than 20% of their body weight within 48 h.

### Proliferation assay

Cell Titer 96^®^ AQueous One Solution Cell Proliferation Assays (MTS) (Promega, Madison, WI) were performed on HTLV-1.wt and HTLV-1.mEnhancer newly immortalized cell lines according to the manufacturer’s protocol. Briefly, cells were counted and plated at 2000 cells/well in 96-well round-bottom plates on day 0 and monitored over a 4-day time course. CellTiter 96^®^ AQueous One Solution reagent was added to each well, agitated slightly, and incubated at 37°C, 5% CO_2_ for 2 hours. Absorbance at 490 nm was collected on a FilterMax F5 Multi-Mode Microplate Reader (Molecular Devices, San Jose, CA). Proliferation in each cell line was measured in triplicate wells at each time point.

### Flow cytometry

Immortalized PBLs (HTLV-1.wt or mEnhancer) were collected by slow centrifugation (5 min, 800 x *g*) for cell phenotype, apoptosis, or cell cycle analysis *via* flow cytometry. Collected cells were stained using fluorescein isothiocyanate (FITC)-conjugated anti-human CD3 and phycoerythrin (PE)-conjugated anti-human CD4 or CD8 antibodies (BD Biosciences, San Jose, CA) and analyzed for cell phenotype by flow cytometry using a Guava^®^ easyCyte™ Benchtop Flow Cytometer. The purity of the isolated CD4^+^ and CD8^+^ T-cell population by positive antibody selection was determined to be between 90-95% using flow cytometry. Percentages of CD4^+^ and CD8^+^ T-cells were determined within the CD3^+^ T-cell gate and were normalized to 100. Alternatively, collected cells were stained using the FITC Annexin V Apoptosis Detection Kit (BD Biosciences) according to the manufacturer’s instructions to measure the level of cellular apoptosis. For cell cycle analysis, 1 x 10^5^ HTLV-1.wt and HTLV-1.mEnhancer immortalized PBLs were seeded in 96-well round-bottom plates. Cells were synchronized by serum starvation for 2 hr. Twenty-four hours later, cells were fixed in 70% ice-cold ethanol for 3 h and stained using the Guava^®^ Cell Cycle Reagent (Luminex Corporation, Austin, TX) according to the manufacturer’s protocol. Data were acquired using the Guava CellCycle program (CytoSoft Version 1.3) and the Guava^®^ easyCyte™ Benchtop Flow Cytometer.

### Rabbit model

Fourteen-week-old, male, specific pathogen-free New Zealand White (052 CR; 571 OAKWOOD) rabbits were obtained from Charles River Laboratories (Wilmington, MA). After a 2-week acclimatization period, 1 x 10^7^ lethally irradiated (100 Gy) 729.B control cells or 729 HTLV-1 producer cells (wt or mEnhancer) were inoculated into the lateral ear vein. Blood was drawn *via* the central auricular artery at Weeks 0 (pre-inoculation), 4, 8, 12, 16, 20, and 25 (study endpoint). Plasma was collected and rabbit PBMCs (rPBMCs) were isolated using Ficoll-Paque™ PREMIUM (Cytiva, Marlborough, MA). rPBMCs or plasma were assessed for proviral load, HTLV-1 gene expression, and HTLV-1-specific antibody response, as described below. Sanger sequencing of the viral enhancer region was performed at week 25 to monitor for viral reversions. All animal procedures were performed in accordance with a protocol approved by University Laboratory Animal Resources (ULAR) of The Ohio State University.

### Quantitative PCR

Genomic DNA and RNA were isolated from cell lines using the AllPrep DNA/RNA Mini Kit (QIAGEN, Hilden, Germany) according to manufacturer’s instructions. RNA samples were subjected to on-column DNase digestion using an RNase-Free DNase (QIAGEN, Hilden, Germany). RNA concentrations were measured using the ND-1000 Nanodrop spectrophotometer (Thermo Fisher Scientific, Waltham, MA), and 250 ng was used for cDNA synthesis using the SuperScript IV First-Strand Synthesis System (Invitrogen, Carlsbad, CA). 2 μL cDNA was used per qPCR reaction with iQ™ SYBR^®^ Green Supermix (Bio-Rad, Hercules, CA) and 300 nM of each sense and antisense primer (20 μL final volume). For Env detection only, 500 ng of RNA was reverse transcribed, and 2 μL cDNA was pre-amplified using the SsoAdvanced™ PreAmp Supermix (Bio-Rad, Hercules, CA). The pre-amplification assay pool included primers for Env and human GAPDH (hGAPDH). The final reaction volume was 50 μL with 50 nM of each primer. The cycling protocol was as follows: 95°C for 3 min followed by 12 cycles of 95°C for 15 s and 58°C for 4 min. Products were diluted 1:5 in TE buffer (pH 8.0, RNase-free; Thermo Fisher Scientific Baltics UAB, Vilnius, Lithuania) for detection of Env and 1:50 for detection of high abundance targets (i.e., hGAPDH), according to the manufacturer’s protocol. Approximately 8.8 μL diluted, pre-amplified products were used per qPCR reaction. Reactions were carried out in 96-well plates on the CFX96 Touch Real-Time PCR Detection System (Bio-Rad, Hercules, CA). The reaction conditions were 50°C for 2 min, 95°C for 10 min, followed by 40 cycles of 15 sec at 95°C and 1 min at 60°C. Primer and probe sequences are listed in **Table 1**. For p12, p30, and p13 detection, 500 ng of RNA was reverse transcribed, and 4 μL cDNA was pre-amplified using the SsoAdvanced™ PreAmp Supermix (Bio-Rad, Hercules, CA). The pre-amplification assay pool included primers for p12, p30, p13, and hGAPDH. The pre-amplification protocol and qPCR reactions were carried out as described above. To determine proviral load, 250 ng genomic DNA was used for qPCR with a primer and probe set specific to HTLV-1 gag/pol. The 20 μL final reaction volume included iQ™ SYBR^®^ Green Supermix (Bio-Rad, Hercules, CA) and 300 nM each of 5’ primer (#20) and 3’ primer (#19). The reaction conditions were 50°C for 2 min, 95°C for 10 min, followed by 40 cycles of 15 sec at 95°C and 1 min at 60°C. Total copy number was calculated using a standard curve generated by duplicate log_10_ dilutions of ACHneo plasmid DNA. Proviral copies per 100 hPBMCs was calculated based on the approximation that 6 pg human DNA is equivalent to 1 cell.

**Table 1:** Primer and probe sequences.

Target	Plasmid Standard	Primers & Probes	Name & Sequence
Gag/pol	ACHneo	5’ primer	[#20] 5′-AGCCCCCAGTTCATGCAGACC-3′
		3’ primer	[#19] 5′-GAGGGAGGAGCAAAGGTACTG-3′
		Probe	[TMP-3] 5′-/56-FAM/CTGCCAAAG/ZEN/ACCTCCAAGACCTCC/3IABkFQ/-3′
Env-4641	ML627	5’ primer	[ENV4641-S] 5′-CGTCCGCCGTCTAGCTTCC-3′
		3’ primer	[ENV-AS] 5′-ATTGTGAGAGTACAGCAGC-3′
Tax	SE356	5’ primer	[X2TR1-2] 5′-ACCAACACCATGGCCCA-3′
		3’ primer	[TR1-AS] 5′-GAGTCGAGGGATAAGGAAC-3′
p13	ML628	5’ primer	[P13-S] 5′-GTCCGCCGTCTAGCAGGT-3′
		3’ primer	[TR-AS] 5′-CCGAACATAGTCCCCCAGAGA-3′
p12-6383	pMT2-2.3	5’ primer	[P12-6383-S] 5′-GTCCGCCGTCTAGCAAC-3′
		3’ primer	[P12-AS] 5′-GGAGAAAGCAGGAAGAGC-3′
p30	pMS9-11.1	5’ primer	[X2P30] 5′-ACCAACACCATGGCACTA-3′
		3’ primer	[H1JA2] 5′-AGGAGCGCCGTGAGCGCAAGT-3′
Hbz-365major	JA662	5’ primer	[HBZMAP1] 5′-CTTCTAAGGATAGCAAACCGTCAAG-3′
		3’ primer	[HBZMAP2] 5′-ATGGCGGCCTCAGGGCT-3′
		Probe	[TMP-13] 5′-/56-FAM/CCTGTGCCA/ZEN/TGCCCGGAGGA/3IABkFQ/-3′
hGAPDH	hGAPDH	5’ primer	[hGAPDH-S] 5′-CATCAATGACCCCTTCATTGAC-3′
		3’ primer	[hGAPDH-AS] 5′-CGCCCCACTTGATTTTGGA-3′
rGAPDH	rGAPDH	5’ primer	[rGAPDH-S] 5′-GATGCTGGTGCCGAGTACGTG-3′
		3’ primer	[rGAPDH-AS] 5′- GTGGTGCAGGATGCGTTGCTGA-3
		Probe	[BY-1Z] 5′-/56-FAM/ACCACCATG/ZEN/GAGAAGGCCGGG/3IABkFQ/-3′
Enhancer region	N/A	5’ primer	[1F] 5’-ACGCGTTATCGGCTCAGC-3’
		3’ primer	[7R] 5’-CTGTATGAGGCCGTGTGA-3’

Genomic DNA and RNA were isolated from rPBMCs and HIS mouse spleens using the AllPrep DNA/RNA Mini Kit (QIAGEN, Hilden, Germany) according to manufacturer’s instructions. RNA samples were subjected to on-column DNase digestion, and 250 ng of RNA was used for cDNA synthesis, as described above. For rabbit samples only, 10 μL cDNA was pre-amplified using the SsoAdvanced™ PreAmp Supermix (Bio-Rad, Hercules, CA). The pre-amplification assay pool included primers for Hbz, gag/pol, and rabbit GAPDH (rGAPDH). The final reaction volume was 50 μL with 50 nM of each primer. The cycling protocol was as follows: 95°C for 3 min followed by 12 cycles of 95°C for 15 s and 58°C for 4 min. Products were diluted 1:5 in TE buffer (pH 8.0, RNase-free; Thermo Fisher Scientific Baltics UAB, Vilnius, Lithuania) for detection of viral genes and 1:50 for detection of high abundance targets (i.e., rGAPDH). Approximately 8.6 μL diluted, pre-amplified products were used per qPCR reaction with iQ™ Supermix (Bio-Rad, Hercules, CA), 300 nM of each sense and antisense primer, and 100 nM probe (20 μL final reaction volume). The reaction conditions were 95°C for 3 min followed by 45 cycles of 95°C for 15 s and 57.5°C for 30 s. Total copy numbers of each gene target in cell lines, rPBMCs, and HIS mice were determined by log_10_ dilutions of plasmid DNA to generate a standard curve. Copy numbers were normalized appropriately to 1 x 10^6^ rabbit or human GAPDH. Samples and standards were run in duplicate with no-RT and no-template controls included on each plate. To determine proviral load, 250 ng genomic DNA was used for qPCR with a primer and probe set specific to HTLV-1 gag/pol. The 20 μL final reaction volume included iQ™ Supermix (Bio-Rad, Hercules, CA), 300 nM each of 5’ primer (#20) and 3’ primer (#19), and 100 nM probe (TMP-3). The reaction conditions were as follows: 94°C for 3 min followed by 45 cycles of 94°C for 15 s, 55°C for 30 s, and 72°C for 40 s. Total copy number was calculated using a standard curve generated by duplicate log_10_ dilutions of ACHneo plasmid DNA. Proviral copies per cell were determined by estimating that 1 μg rPBMC DNA is equivalent to 134,600 cells (24). Analysis of Gag/pol and Hbz copy numbers normalized to proviral load in rabbits was performed by first normalizing total copy number of each gene target to 1 x 10^6^ rGAPDH and then normalizing to proviral copies per cell. Proviral copies per 100 hPBMCs in HIS mice was calculated based on the approximation that 6 pg human DNA is equivalent to 1 cell. Analysis of Tax and Hbz copy numbers normalized to proviral load in HIS mice was performed by first normalizing total copy number of each gene target to 1 x 10^6^ hGAPDH and then normalizing to proviral copies per 100 hPBMCs.

### Sequencing

Sequencing was performed to verify enhancer mutations in 729 HTLV-1.mEnhancer producer cells and to screen for mutation reversions in PBL cell lines from *in vitro* immortalization assays, as well as in infected rabbits and HIS mice. Genomic DNA was isolated from producer and PBL cell lines using the DNeasy Blood & Tissue Kit (QIAGEN, Hilden, Germany). Genomic DNA was isolated from rPBMCs at the study endpoint (Week 25) and hPBMCs collected from mouse spleen at the time of sacrifice (varied for individual mice infected with HTLV-1.wt or HTLV-1.mEnhancer) using the AllPrep DNA/RNA Mini Kit (QIAGEN, Hilden, Germany). The mEnhancer region was amplified by PCR using GoTaq^®^ Flexi DNA Polymerase (Promega, Madison, WI) and the following flanking primers: 1F and 7R. The PCR conditions were as follows: 95°C for 2 min followed by 95°C for 30 s, 52.2°C for 30 s, and 72°C for 1 min (35 cycles), and a final extension of 72°C for 5 min. PCR products were run on a 1.5% TAE agarose gel with ethidium bromide. The amplified fragment was gel extracted using the QIAquick Gel Extraction Kit (QIAGEN, Hilden, Germany) and Sanger sequencing was performed with individual reactions containing forward or reverse primer (OSUCCC Genomics Shared Resource). Sequencing data was analyzed using SnapGene^®^ 5.1.5 software (GSL Biotech LLC, San Diego, CA).

### Rabbit antibody ELISA

The HTLV-specific antibody response was measured using the Avioq HTLV-I/II Microelisa System (Avioq, Inc., Research Triangle Park, NC). The manufacturer’s protocol was modified by substituting the provided HRP-conjugated goat anti-human IgG with an HRP-conjugated goat anti-rabbit IgG (ab6721; Abcam, Cambridge, United Kingdom). Rabbit plasma was diluted 1:50 with Sample Diluent (supplied by the manufacturer) to obtain absorbance values within the range of the standard curve. Absorbance was measured at 450 nm using the FilterMax F5 Multi-Mode Microplate Reader (Molecular Devices, San Jose, CA).

### Statistical analyses

Data were analyzed using the appropriate statistical test (denoted in figure legend) with GraphPad Prism 9.0 software (GraphPad Software Inc., La Jolla, CA).

## Results

### Generation of HTLV-1.mEnhancer proviral clone and producer cells

A novel HTLV-1 enhancer was recently identified within the HTLV-1 provirus through a screen for nucleosome-free regions (NFR), which generally harbor transcriptional regulatory elements due to their accessibility to transcription factors (19). Matsuo et al. found a region highly depleted of nucleosomes located between the viral insulator, containing a CCCTC-binding factor (CTCF) binding site, and the 3’ LTR (19). Luciferase reporter assays in Jurkat cells demonstrated that 3’ LTR promoter activity was enhanced by the presence of the NFR, whether it was inserted upstream or downstream of the promoter in either a sense or antisense orientation, relative to 3’ LTR activity alone. Further analyses demonstrated that the NFR contained histone markers characteristic of enhancer elements, including H3K4 methylation and H3K27 acetylation, and that the region produces bi-directional, capped RNAs, a feature of transcription at active enhancers (25–27). Evaluation of candidate binding factors revealed that the transcription factors SRF and ELK-1, which form a ternary complex at serum response elements (28–31), exhibit sequence-specific binding to the NFR, and that mutation of the SRF/ELK-1 binding sites resulted in decreased enhancer activity in transfected cells. To determine the effect of the viral enhancer on HTLV-1-mediated *in vitro* immortalization as well as *in vivo* persistence and pathogenesis, we introduced mutations into the SRF/ELK-1 binding sites using the established wild-type (wt) HTLV-1 molecular clone ACH (HTLV-1.wt) to generate HTLV-1.mEnhancer (**Figure 1A**). Although the mEnhancer region overlaps with exon 3 of the Tax gene, the nucleotide substitutions do not alter the amino acid sequence of Tax. To determine whether HTLV-1.mEnhancer retained Tax transcriptional activity, we co-transfected the HTLV-1.mEnhancer or the HTLV-1.wt proviral clone with an LTR-1-Luc reporter and a TK-Renilla transfection control into HEK293T cells. HTLV-1.mEnhancer displayed no significant difference in LTR-driven luciferase activity compared to the HTLV-1.wt (**Figure 1B**) and levels of p19 Gag produced by transfected cells were similar (**Figure 1C**).

Previously, mutation of the HTLV-1 enhancer element resulted in lower levels of both sense and antisense viral transcripts (19). Therefore, we measured both *tax* (sense) and (*hbz*) (antisense) mRNA transcript levels in transfected HEK293T cells. Copy numbers of viral genes were normalized to 1 x 10^6^ human *gapdh* (hgapdh) copies in each sample. While there was no difference in the copy number of *tax* between the HTLV-1.wt and HTLV-1.mEnhancer proviral clones, HTLV-1.mEnhancer showed a slight (however not significant) reduction in *hbz* (**Figure 1D**). To measure viral protein expression, cell lysates were subjected to immunoblotting. Tax protein was detectable, although at reduced levels, in cells expressing the HTLV-1.mEnhancer compared to HTLV-1.wt (**Figure 1E**). While this appears contradictory to the copy numbers of Tax detected by qPCR in the wt and mutant conditions, even a small reduction in mRNA levels could correspond to a decrease in protein expression. Hbz protein could not be detected, which is likely a reflection of the differences in copy number between this antisense transcript and Tax. To assess the effect of the enhancer element in CD4^+^ T-cells, the primary target of HTLV-1 infection and virus-mediated immortalization, we conducted luciferase reporter assays in Jurkat cells, an HTLV-1-negative, CD4^+^ T-cell line. Although the transfection efficiency in Jurkat cells was much lower compared to HEK293T cells, there was no difference in LTR-driven gene expression (**Figure 1F**) or p19 Gag production (**Figure 1G**) between HTLV-1.wt and the HTLV-1.mEnhancer.

**Figure 2:**
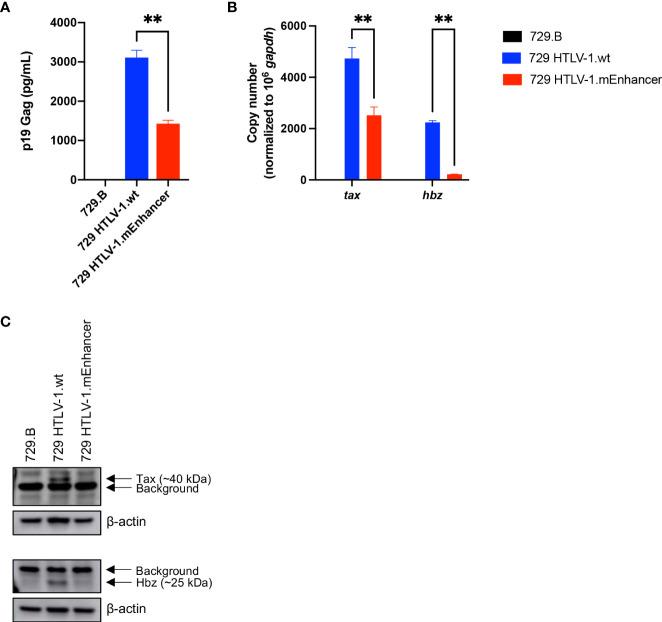
Generation and characterization of HTLV-1.mEnhancer producer cells. HTLV-1.wt and HTLV-1.mEnhancer producer cells were generated by nucleofecting 729.B parental cells with proviral plasmid DNA. After G418 selection and limiting dilution, **(A)** supernatant was collected from single cell clones for p19 Gag ELISA. Only one clone with sufficient virion production compared to HTLV-1.wt was selected for additional experiments and analyses. Statistical significance was determined by unpaired t test. **P ≤ 0.01. **(B)** RNA was extracted from the HTLV-1.mEnhancer clone for cDNA synthesis and qPCR to detect *tax* and *hbz* mRNA expression. Statistical significance was determined by two-way ANOVA. **P ≤ 0.01. **(C)** Total protein in cell lysates from parental 729.B cells, 729 HTLV-1.wt, and 729 HTLV-1.mEnhancer producer cells was quantified, and equal amounts were loaded onto an SDS-PAGE gel for immunoblotting analysis. β-actin is shown as a loading control, and arrows differentiate background from bands that represent Tax and Hbz protein. Graphs represent data generated from duplicate samples and error bars represent standard deviation (SD).

Given that cell-to-cell contact is required for efficient transmission of HTLV-1 (32–35), we generated a stable producer cell line carrying the HTLV-1.mEnhancer proviral clone to characterize the effects of the enhancer element on *in vitro* immortalization and *in vivo* persistence and disease development. Following the introduction of proviral DNA into 729.B parental cells, transfectants were subjected to antibiotic selection and limiting dilution to isolate stable cell clones. The presence of enhancer mutations in the SRF and ELK-1 binding sites was confirmed in single cell clones by sequencing (data not shown). The 729 HTLV-1.mEnhancer cells had significantly lower p19 Gag in the culture supernatant (**Figure 2A**), although this could partly be attributed to the copy number and location of viral sequence within the cellular genome. The mutant cell line with the highest level of virion production was used for further analyses and normalized for p19 Gag, relative to the previously established 729 HTLV-1.wt clone (36). 729 HTLV-1.mEnhancer cells had significantly reduced *tax* and *hbz* mRNA expression (**Figure 2B**) and a corresponding reduction in the protein expression of these viral genes (**Figure 2C**) compared to the HTLV-1.wt producer cells. Overall, our results in HEK293T and stable 729 cell transfectants show that the HTLV-1 internal enhancer contributes to increased viral transcription *in vitro*.

### The HTLV-1 internal enhancer is dispensable for early viral persistence *in vivo*

**Figure 3:**
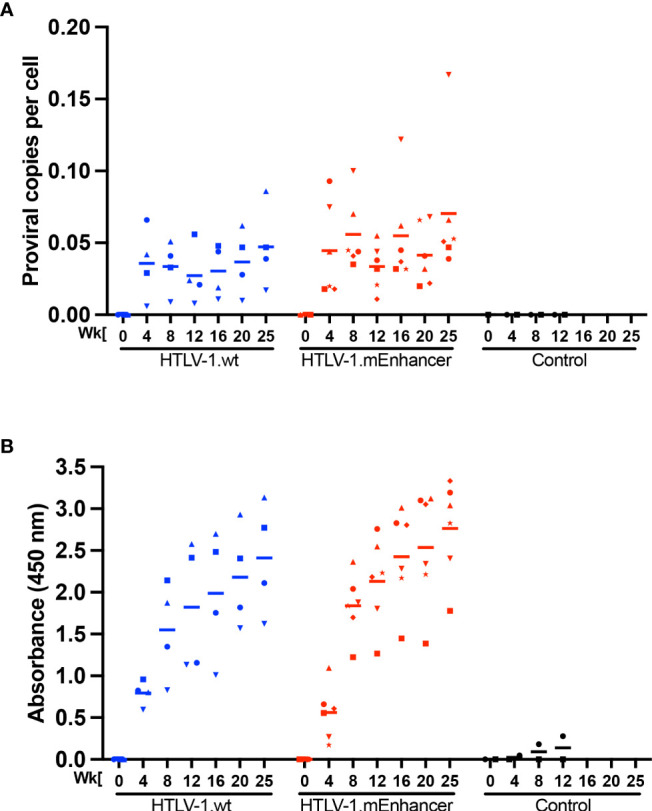
HTLV-1 enhancer element is dispensable for early *in vivo* viral persistence. Lethally irradiated 729 HTLV-1.wt or 729 HTLV-1.mEnhancer producer cells were inoculated into 14-week-old, male New Zealand white rabbits *via* the lateral ear vein. Blood was collected at Week 0 (pre-inoculation) and Weeks 4, 8, 12, 16, 20, and 25 post-infection (study endpoint) for plasma and rabbit PBMC (rPBMC) isolation. **(A)** Genomic DNA was isolated from rPBMCs and subjected to qPCR to detect proviral load using a primer and probe set specific to HTLV-1 Gag/pol. **(B)** Plasma was isolated from whole blood to measure the HTLV-specific antibody response using the Avioq HTLV-I/II Microelisa System. Absorbance was measured at 450 nm. In each of the graphs, unique symbols represent proviral load and antibody response for a single inoculated rabbit over time and bars represent the mean. Linear mixed-effects analyses were performed and multiple comparisons were adjusted by Tukey’s method.

**Figure 4:**
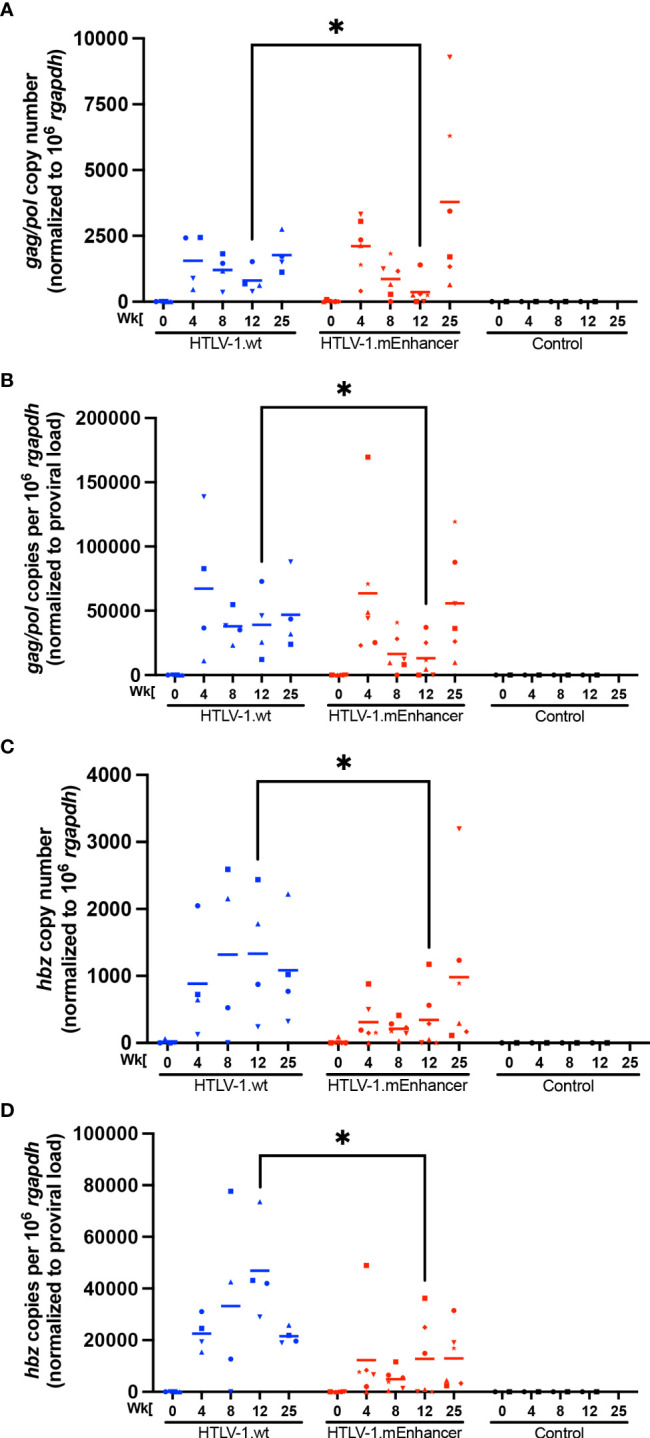
HTLV-1 enhancer element alters *in vivo* sense and anti-sense viral gene expression. RNA was isolated from rPBMCs for cDNA synthesis. cDNA from rabbit samples and negative controls was used in 12-cycle pre-amplification reactions. Pre-amplification products were diluted according to the manufacturer’s directions for qPCR to detect viral gene expression. Copy numbers of Gag/pol **(A)** and Hbz **(C)** are shown relative to 1 x 10^6^ *rgapdh* copies. The levels of viral transcripts were evaluated by normalizing Gag/pol **(B)** or Hbz **(D)** copies per 10^6^ *rgapdh* to proviral load. In each of the graphs, unique symbols represent gene expression for a single inoculated rabbit over time and bars represent the mean. Linear mixed-effects analyses were performed and multiple comparisons were adjusted by Tukey’s method. *P ≤ 0.05.

To evaluate the role of the viral enhancer on the early events of HTLV-1 infection *in vivo*, we utilized the previously characterized, immune competent NZW rabbit model of HTLV-1 replication and persistence (18, 24, 37–41). Rabbits were inoculated with lethally irradiated 729 HTLV-1.wt or 729 HTLV-1.mEnhancer producer cells, and blood was drawn at various time points over the course of a 25-week study. qPCR was used to detect HTLV-1 gag/pol DNA sequence beginning at Week 4 post-infection. Proviral copies per cell were variable in individual rabbits at each time point, but generally increased throughout the study (**Figure 3A**). There were no significant differences in proviral load between the rabbits infected with HTLV-1.mEnhancer compared to HTLV-1.wt. Sequencing of genomic DNA isolated from rabbit PBMCs (rPBMCs) collected at the study endpoint (Week 25) confirmed the presence of the expected mutations in rabbits infected with HTLV-1.mEnhancer (data not shown). To determine whether HTLV-1.mEnhancer could elicit a virus-specific immune response similar to rabbits infected with HTLV-1.wt, plasma was isolated from whole blood samples and analyzed *via* a Microelisa system for the detection of HTLV-1/2 antibodies. As previously shown, the anti-HTLV-1 response rose throughout the study but was variable in individual rabbits (18). Rabbits in both the wt and mEnhancer groups had seroconverted by Week 4, and the HTLV-1-specific antibody response increased over time with no significant difference in the level of the response between groups (**Figure 3B**). Given the role of the HTLV-1 enhancer in the transcription of viral genes *in vitro*, we assessed whether mutation of the transcription factor binding sites within the enhancer affected viral gene expression *in vivo*. RNA isolated from rPBMCs at various time points was reverse transcribed, pre-amplified using primers specific to viral genes, and quantified by probe-based qPCR. Viral copy numbers were normalized to 1 x 10^6^ rabbit *gapdh* (rgapdh) copies in each sample. Tax was undetectable in most rabbits from both the wt and mutant groups, and it was previously demonstrated that *tax* mRNA expression peaks as early as 1-2 weeks post-infection (24, 39), and that the levels of this viral transcript are at the limit of detection of qPCR (37). Transcript for *gag/pol* was therefore used as a measure of viral sense transcription. *Gag/pol* copy number was variable at each time point but was highest by Week 25 (**Figure 4A**). There was a significant decrease in *gag/pol* transcript level in the HTLV-1.mEnhancer infected rabbits compared to HTLV-1.wt infected rabbits at week 12. Interestingly, PBMCs from two rabbits infected with HTLV-1.mEnhancer showed *gag/pol* levels that were much higher compared to the other rabbits from both conditions at week 25. However, there was no significant difference in mean *gag/pol* expression between HTLV-1.wt and HTLV-1.mEnhancer at Week 25 *in vivo*. The rabbit in the HTLV-1.mEnhancer condition with the highest level of *gag/pol* expression in infected PBMCs at Week 25 also had the highest proviral load at this time point (inverted triangle). To assess viral transcription levels according to the proviral copies per cell, *gag/pol* copy number was also normalized to the respective proviral load in each rabbit. Rabbits infected with HTLV-1.wt and HTLV-1.mEnhancer showed similar trends in the levels of *gag/pol* transcript per proviral copy number over the course of time, with the only statistically significant difference at Week 12 (**Figure 4B**). Hbz was also a gene product of interest, given its role in promoting persistent infection *in vivo* (18). The level of *hbz* mRNA increased over time in animals infected with HTLV-1.wt compared to HTLV-1.mEnhancer and at week 12 there was a statistically significant decrease in *hbz* in the HTLV-1.mEnhancer infected rabbits (**Figure 4C**). Therefore, *hbz* mRNA expression was delayed in the HTLV-1.mEnhancer rabbits early to midway through the study, but the mean expression rose to the level of the HTLV-1.wt group by Week 25. The level of *hbz* transcript was also highest in the HTLV-1.mEnhancer rabbit with the highest proviral load (inverted triangle); therefore, *hbz* copy number was normalized to proviral load in each animal. The level of *hbz* copies per proviral copy number was significantly lower in the HTLV-1.mEnhancer condition, but by Week 25, the expression was comparable to the level of HTLV-1.wt infected rabbits (**Figure 4D**). This likely reflects the importance of *Hbz* in the establishment and maintenance of viral persistence in the presence of a functional immune system in the rabbits. Fluctuations in the levels of *gag/pol* and *hbz* mRNA in individual rabbits at different time points may also be due to changes in clone predominance throughout the study. Taken together, our data show that the intragenic enhancer of HTLV-1 is dispensable for viral persistence, however, the enhancer element does influence viral gene transcription at varying time points after infection.

### Mutation of the HTLV-1 enhancer has no effect on leukemogenesis *in vivo*

**Figure 5:**
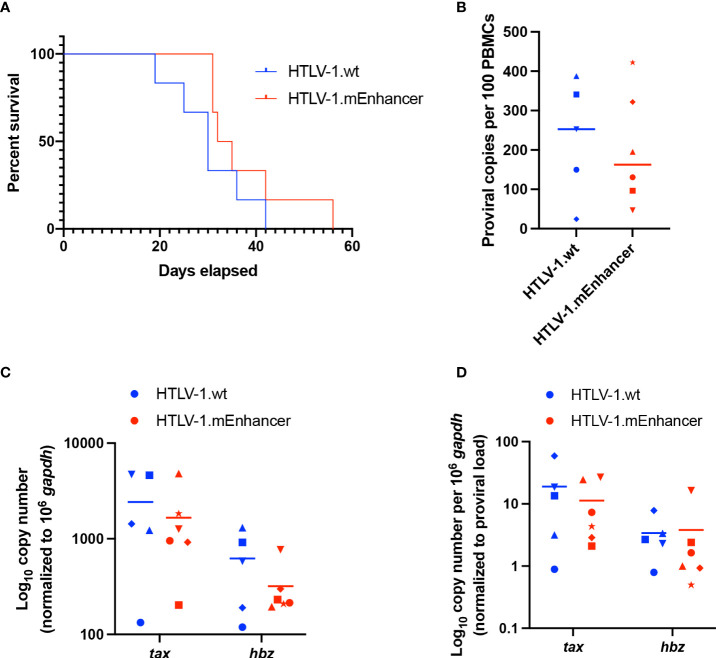
Loss of enhancer element has no effect on disease progression in HIS mice. Sub-lethally irradiated neonatal NSG received liver injections of 3 x 10^4^ to 1 x 10^5^ of CD34^+^ HUSC. 10 weeks after HUSC engraftment, mice were inoculated intraperitoneally with 1 x 10^7^ lethally irradiated 729 HTLV-1.wt or 729 HTLV-1.mEnhancer producer cells. **(A)** HTLV-1 infection induces lymphoproliferative disease in the mice, and survival rate was determined for animals inoculated with wt compared to mEnhancer virus. Mice were euthanized according to early removal criteria defined in the approved animal protocol. Statistical significance was determined by Log-rank (Mantel-Cox) test. **(B)** Genomic DNA was extracted from PBMCs isolated from mouse spleens and used for qPCR to detect proviral load using primers targeting HTLV-1 Gag/pol. Statistical significance was determined by Welch’s unpaired t test. **(C)** RNA extracted from hPBMCs was subjected to cDNA synthesis followed by qPCR to detect viral gene expression. Data are shown normalized to 1 x 10^6^ *hgapdh* copies. **(D)** The levels of viral transcripts were evaluated by normalizing Tax or Hbz copies per 10^6^ *hgapdh* to proviral load. Unique symbols in **(B–D)** represent proviral load, gene expression, and viral transcripts per proviral copy number, respectively, in a single inoculated mouse and bars represent the mean. Statistical significance was determined by unpaired t-test.

To determine the effect of the viral enhancer on HTLV-1-mediated disease development, we inoculated lethally irradiated virus producer cells into humanized immune system (HIS) mice, which were previously shown to be susceptible to lymphoproliferative disease induced by infectious HTLV-1 molecular clones (42). Sub-lethally irradiated neonatal NSG mice were injected with CD34^+^ human umbilical cord stem cells and monitored for the development of mature lymphocyte populations. In these mice, phenotypically normal human lymphocytes develop which are unable to mount adaptive immune responses. Mice were continually evaluated for weight loss and additional clinical signs of virus-induced lymphoproliferative disease. There was no significant difference in the survival percentage of mice infected with HTLV-1.wt compared to HTLV-1.mEnhancer; animals in each group became moribund as the study progressed and had to be euthanized (**Figure 5A**). Spleens were collected from euthanized mice for isolation of human PBMCs (hPBMCs), followed by genomic DNA and RNA extraction. Proviral copies were detected by qPCR using primers specific to HTLV-1 gag/pol. While the mean proviral load in the HTLV-1.mEnhancer-infected group was reduced compared to mice infected with HTLV-1.wt, the difference was not statistically significant (**Figure 5B**). Sequencing of integrated proviruses from HIS mouse genomic DNA confirmed the presence of the expected mutations in mice infected with HTLV-1.mEnhancer (data not shown). RNA was reverse transcribed and viral sense and antisense transcripts were quantified by detecting *tax* and *hbz* mRNA levels, respectively. Tax expression was similar in hPBMCs infected with HTLV-1.wt or HTLV-1.mEnhancer (**Figure 5C**). Hbz mRNA expression in the HTLV-1.mEnhancer group trended lower compared to the HTLV-1.wt group, but there was no statistically significant difference (**Figure 5C**). Tax and Hbz transcripts were also analyzed by normalizing to the proviral load in each respective animal. There were no significant differences in *tax* or *hbz* mRNA copies normalized to proviral load between the HTLV-1.wt and HTLV-1.mEnhancer conditions (**Figure 5D**). Overall, our analyses indicate that HTLV-1.mEnhancer could replicate and induce disease progression in the HIS mouse model.

### HTLV-1.mEnhancer immortalizes primary human T-cells in culture

**Figure 6:**
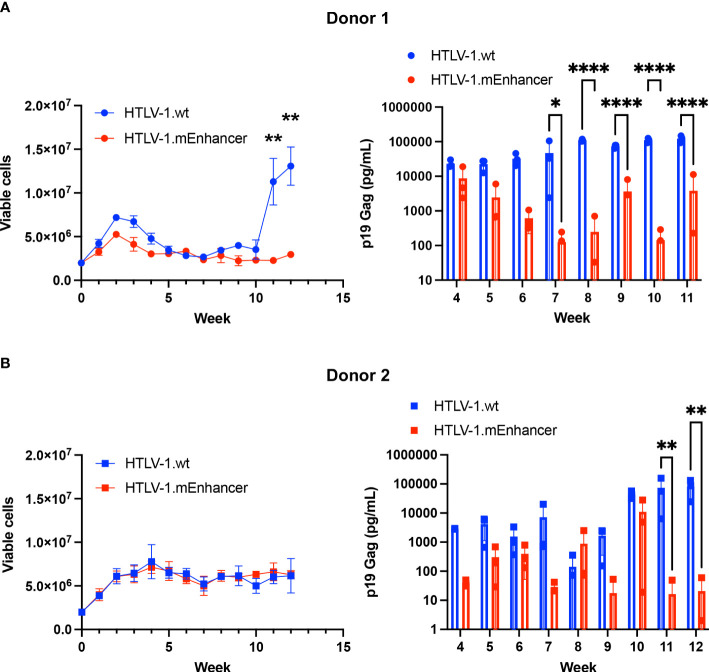
HTLV-1.mEnhancer virus immortalizes primary human T-cells *in vitro*. Freshly isolated human PBMCs (2 x 10^6^; hPBMCs) from two healthy donors were co-cultured independently with 1 x 10^6^ lethally irradiated 729 HTLV-1.wt or HTLV-1.mEnhancer producer cells in 24-well plates for long-term immortalization assay. Cells were supplied with 10 U/mL hIL-2 once per week with media changes. T-cell immortalization in the co-culture with PBMCs from Donor 1 **(A)** and Donor 2 **(B)** was determined by weekly viable cell counts by trypan blue exclusion. Cell supernatant was collected at weekly intervals, beginning at Week 4, to measure virion production by p19 Gag ELISA. Samples from Donor 1 are represented by circles, and samples from Donor 2 are represented by squares. Each time point depicts data collected from three random, independent wells (technical replicates), and error bars represent SD. Wells with a p19 Gag value of zero are not shown in the graph due to log transformation. Statistical significance was determined by unpaired t test and two-way ANOVA with Sidak’s multiple comparisons test, where appropriate. *P ≤ 0.05, ** P ≤ 0.01, ****P ≤ 0.0001.

To determine the effect of the mEnhancer on HTLV-1-mediated immortalization of primary cells, we co-cultured human PBMCs freshly isolated from healthy donors with lethally irradiated 729 HTLV-1.wt or 729 HTLV-1.mEnhancer producer cells in two independent co-culture experiments. 10 U/ml human IL-2 was supplied weekly to the culture with media changes. Over time, both HTLV-1.wt and HTLV.1-mEnhancer viruses demonstrated the capacity to immortalize T-cells. At 10 weeks, cells from Donor 1 infected with HTLV-1.wt showed rapid proliferation as individual wells began to grow out from the co-culture as an immortalized, polyclonal cell population. Cells infected with HTLV-1.mEnhancer exhibited the typical cell clumping that is observed with immortalized PBL lines but grew at a much slower rate in culture (**Figure 6A**). PBMCs co-cultured with HTLV-1-negative 729.B parental cells failed to sustain long-term proliferation (data not shown). As additional controls, aliquots of lethally irradiated 729 HTLV-1.wt and 729 HTLV-1.mEnhancer producers were maintained in culture to confirm cell death. Further, PBMCs alone were plated and confirmed a lack of progressive cell growth in the absence of HTLV-1 infection. Beginning at Week 4 of the co-culture, cell supernatants were collected from each condition for p19 Gag ELISA. Cells infected with HTLV-1.wt showed an increase in p19 Gag concentration over time, demonstrating continued viral replication and virion production. Levels of p19 Gag production by the HTLV-1.mEnhancer infected cells began to significantly decrease compared to cells infected with wt virus beginning at Week 7 of the co-culture with Donor 1 (**Figure 6A**). Cells from Donor 2 infected with HTLV-1.wt or HTLV-1.mEnhancer showed similar viable cell counts over the 12-week time course, but with a 3-4 week lag in outgrowth of polyclonal cell populations (outgrowth of infected cells occurred after Week 12) (**Figure 6B**). A statistically significant decrease in the levels of p19 Gag production by cells infected with HTLV-1.mEnhancer from Donor 2 was observed by Week 11 of co-culture. HTLV-1.wt and HTLV-1.mEnhancer viruses immortalized PBMCs from each donor with different efficiencies (Donor 1: 11 wt cell lines, 5 mEnhancer cell lines; Donor 2: 4 wt cell lines, 9 mEnhancer cell lines); however, as newly immortalized cell lines were established from each co-culture, there were similarities between donors in the slower proliferation rate of HTLV-1.mEnhancer infected cells compared to those infected with HTLV-1.wt.

**Figure 7:**
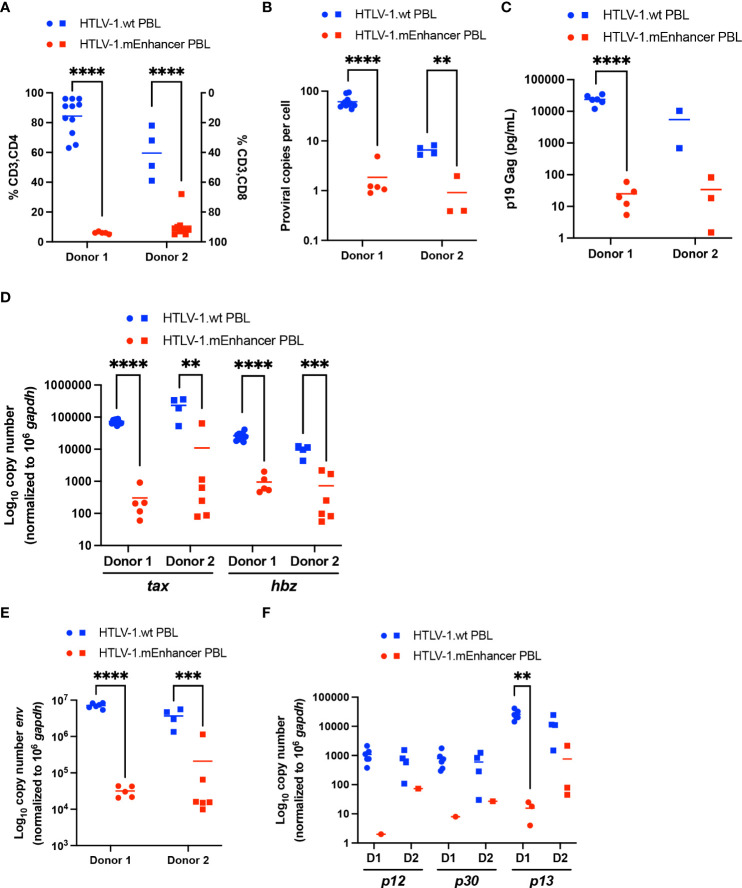
Loss of HTLV-1 enhancer element alters immortalization phenotype and viral gene expression *in vitro*. Newly immortalized PBL cell lines from two separate co-culture experiments with different PBMC donors were characterized *in vitro*. Cell lines from Donor 1 are represented by circles, and cell lines from Donor 2 are represented by squares. **(A)** T-cell phenotypic analysis of HTLV-1.wt (Donor 1 n=11; Donor 2 n=4) or HTLV-1.mEnhancer PBLs (Donor 1 n=5; Donor 2 n=9) was performed by flow cytometry. **(B)** Genomic DNA was extracted from PBL cell lines and used for qPCR to detect proviral load using primers targeting HTLV-1 Gag/pol (HTLV-1.wt: Donor 1 n=11, Donor 2 n=4; HTLV-1.mEnhancer: Donor 1 n=5, Donor 2 n=3). **(C)** Approximately 5 x 10^5^ PBLs were plated in 1 mL media. Supernatant was collected from each well after 72h and analyzed by p19 Gag ELISA. (HTLV-1.wt: Donor 1 n=6, Donor 2 n=2; HTLV-1.mEnhancer: Donor 1 n=5, Donor 2 n=3) **(D)** RNA was extracted from each PBL cell line, and subjected to cDNA synthesis followed by qPCR to detect *tax* and *hbz* gene expression. Data are shown normalized to 1 x 10^6^ *hgapdh* copies (HTLV-1.wt: Donor 1 n=11, Donor 2 n=4; HTLV-1.mEnhancer: Donor 1 n=5, Donor 2 n=6) **(E, F)** PBL RNA was isolated and used for cDNA synthesis. Pre-amplification was performed, followed by qPCR to determine expression of *env*, *p12*, *p30*, and *p13* viral transcripts. Data are shown normalized to 1 x 10^6^ *hgapdh* copies. Values of zero for cell lines with undetectable transcripts are not plotted or included in the statistical analyses (HTLV-1.wt: Donor 1 n=6, Donor 2 n=4; HTLV-1.mEnhancer: Donor 1 n=5, Donor 2 n=6). Donor 1 and Donor 2 are abbreviated as D1 and D2, respectively, in **(E)**. Statistical significance was determined by unpaired t test. **P ≤ 0.01, ***P ≤ 0.001, ****P ≤ 0.0001.

Newly immortalized HTLV-1.wt and HTLV-1.mEnhancer PBL cell lines established from co-culture experiments with two different blood donors were phenotyped for CD3, CD4, and CD8 surface expression. While HTLV-1 can infect a wide variety of hematopoietic cells, the virus preferentially immortalizes CD4^+^ T-cells *in vitro*, and ATL and HAM/TSP are CD4^+^ T-cell-mediated diseases (24, 43, 44). Using flow cytometry, HTLV-1.wt immortalized T-cells were found to be predominantly CD3^+^CD4^+^, while HTLV-1.mEnhancer immortalized T-cells were overwhelmingly CD3^+^CD8^+^ tropic (**Figure 7A**). This result was consistent between different PBMC donors. Sequencing of genomic DNA isolated from each individual PBL cell line confirmed the presence of the expected mutations within HTLV-1.mEnhancer immortalized cells (data not shown).

HTLV-1.wt and HTLV-1.mEnhancer PBL cell lines were collected and genomic DNA and RNA was extracted. Proviral copies were detected by qPCR using primers specific to HTLV-1 gag/pol. The mean proviral load in the HTLV-1.mEnhancer immortalized group was significantly lower compared to HTLV-1.wt immortalized cells (**Figure 7B**). While the HTLV-1.wt immortalized cells on average had many proviral integrations (~50 for Donor 1; ~8-9 for Donor 2), the HTLV-1.mEnhancer immortalized cells typically featured ~1 proviral integrations. In accordance with the distinct lower number of proviral insertion sites, the HTLV-1.mEnhancer immortalized cells had lower p19 Gag in the culture supernatant (**Figure 7C**).

To measure viral transcription in the newly immortalized cells, isolated RNA was reverse transcribed and viral sense and antisense transcripts were quantified by detecting *tax* and *hbz* mRNA levels, respectively. *Tax* and *hbz* mRNA transcript level was significantly higher in hPBMCs immortalized with HTLV-1.wt compared to HTLV-1.mEnhancer (**Figure 7D**). The observed differences in the copy numbers of *tax* and *hbz* transcripts, as well as p19 Gag levels in the culture supernatant (**Figure 7C**), are greater than what could be solely accounted for by the difference in proviral load between HTLV-1.wt and HTLV-1.mEnhancer infected cells. Previous studies found that the HTLV-1 immortalization phenotype is dictated through the Env region (24). Chimeric proviruses which replaced HTLV-1 Env with the highly similar, but non-pathogenic HTLV-2 Env gene resulted in a transformation switch from predominantly CD4^+^ to CD8^+^ T-cells *in vitro*. This phenomenon was independent of viral entry as both HTLV-1 and HTLV-2 are able to infect both CD4^+^ and CD8^+^ T-cells *in vitro* and *in vivo*. Env transcript in the newly immortalized PBLs was at the limit of qPCR detection, therefore RNA isolated from PBLs was reverse transcribed, pre-amplified using primers specific to viral genes, and quantified by qPCR. Although *env* transcript could be detected in both HTLV-1.wt and HTLV-1.mEnhancer immortalized cells, the level of *env* was significantly lower in mutant compared to wt cells (**Figure 7E**). Given the effects of enhancer disruption on numerous viral transcripts, the expression of other HTLV-1 regulatory genes, including p12, p30, and p13, was also examined. The p12 and p30 transcripts were reduced in cells immortalized by HTLV-1.mEnhancer, however, these transcripts could not be detected in some cell lines due to their low overall abundance even in HTLV-1.wt cells. The regulatory gene p13 was more abundant in both HTLV-1.wt and HTLV-1.mEnhancer PBLs and this transcript was lower in cells infected with mutant virus (**Figure 7F**). Overall, the data suggest that the internal enhancer element is important for efficient transcription from the HTLV-1 provirus in stably infected cells.

**Figure 8:**
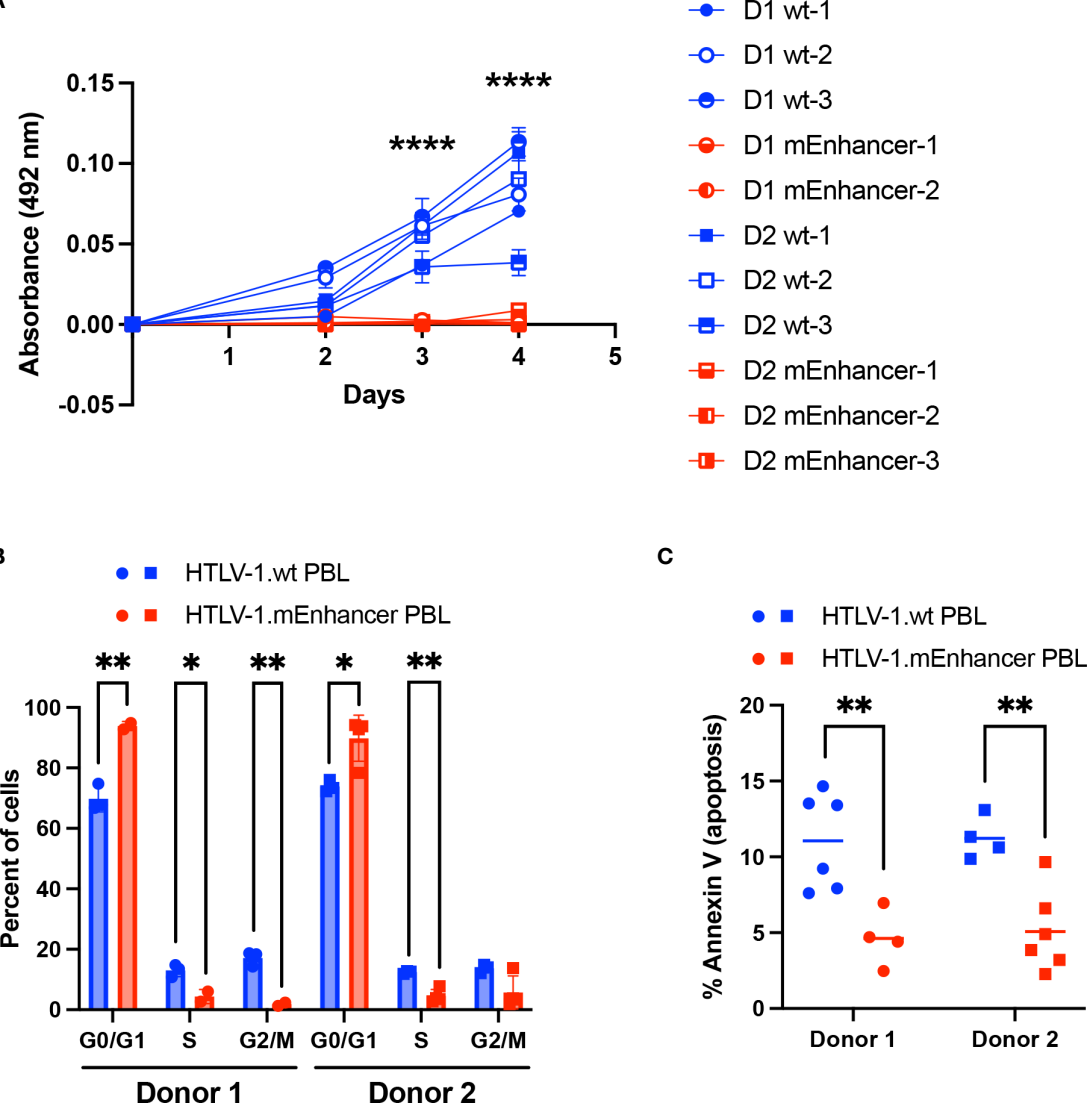
Loss of the HTLV-1 enhancer element leads to reduced proliferation and altered cell cycle progression of infected cells *in vitro*. **(A)** Proliferation of newly immortalized PBL lines was measured by MTS assay. Cell lines from Donor 1 are represented by circles, and cell lines from Donor 2 are represented by squares (HTLV-1.wt: Donor 1 n=3, Donor 2 n=3; HTLV-1.mEnhancer: Donor 1 n=2, Donor 2 n=3). Error bars represent the SD of 3 technical replicates. **(B)** PBL cell lines (HTLV-1.wt: Donor 1 n=3, Donor 2 n=3; HTLV-1.mEnhancer: Donor 1 n=3, Donor 2 n=2) were fixed and stained with propidium iodide, and cell cycle progression was measured by flow cytometry. Bars represent the mean with SD. **(C)** Cellular apoptosis was measured in PBL cell lines (HTLV-1.wt: Donor 1 n=6, Donor 2 n=4; HTLV-1.mEnhancer: Donor 1 n=4, Donor 2 n=6) using a FITC Annexin V Apoptosis Detection Kit. Bars represent the mean. Statistical significance was determined by unpaired t test. *P ≤ 0.05, **P ≤ 0.01, ****P ≤ 0.0001.

While both HTLV-1.wt and HTLV-1.mEnhancer had the capacity to immortalize T-cells *in vitro*, PBL cell lines established in co-culture with mutant producer cells demonstrated slower growth compared to cell lines immortalized by wt virus. To confirm this difference, cell proliferation was measured by MTS assay. HTLV-1.mEnhancer PBL cell lines demonstrated significantly reduced proliferation compared to HTLV-1.wt PBL cell lines at Days 3 and 4 after plating equivalent numbers of cells per condition (**Figure 8A**). These results suggested that cells infected with HTLV-1.mEnhancer may have altered cell cycle progression. Flow cytometry analysis of synchronized PBL cell lines from both donors showed a significantly higher percentage of HTLV-1.mEnhancer immortalized cells in the G0/G1 phase compared to HTLV-1.wt cells. Proportionally, significant decreases in the S and G2/M phases were observed in the HTLV-1.mEnhancer cell population from Donor 1 (**Figure 8B**). Donor 2 PBL lines immortalized by the enhancer mutant also showed a significant decrease in the percentage of cells in S phase compared to wt cells. Since cells infected with HTLV-1.mEnhancer showed altered cell cycle progression and reduced proliferation, we then examined whether these differences were associated with increased cell death. Given that Hbz was also previously reported to confer an anti-apoptotic phenotype in Jurkat cells (45), we predicted that HTLV-1.mEnhancer immortalized cells would have more apoptosis than HTLV-1.wt immortalized cells due to lower levels of *hbz* mRNA. However, HTLV-1.mEnhancer cells had significantly less apoptosis than their wt counterparts from the same blood donors (**Figure 8C**).

## Discussion

The synthesis of HTLV-1 sense gene products, in particular Tax, drives cellular transformation and leukemogenesis through the mechanisms by which viral proteins deregulate cellular pathways and incite genomic instability (8). Despite its high immunogenicity and silenced expression in the majority of ATL cases (46, 47), Tax exerts its functions by alternative modalities now recognized in the field, including transcriptional bursts and sporadic on/off switching in subpopulations of leukemic cells (14, 48, 49). The HTLV-1 antisense gene, Hbz, functions antagonistically to Tax by competing for interaction with the same factors to suppress transcription from the 5’ LTR (17). Hbz expression is continuous in cell culture and in patient cells (10, 17, 47), and it has been shown that Hbz enhances viral persistence *in vivo* (18) and promotes *in vitro* T-cell proliferation in both its mRNA and protein forms (9, 10). Additionally, in the absence of a cytotoxic T-cell response against *hbz* RNA (17), infected cells are able to sustain dysregulated growth and survival. This complex proviral transcription pattern is the key to HTLV-1 pathogenesis.

Recently identified DNA elements within the HTLV-1 provirus that have potential roles in the regulation of sense versus antisense viral gene expression, as well as the surrounding host genome, include the viral enhancer (19) and the insulator region containing the CTCF-binding site (vCTCF-BS) (50). *In vitro* studies on the vCTCF-BS have evaluated its effects on viral and host gene transcription, chromatin organization, and epigenetic modifications (50–52). A subsequent study utilized co-culture assays and the NZW rabbit model to reveal that the vCTCF-BS is not required for *in vitro* T-cell immortalization or *in vivo* HTLV-1 persistence (37). Given that the enhancer is located between the vCTCF-BS and the 3’LTR in a region highly depleted of nucleosomes, we sought to characterize the newly discovered viral DNA element in the context of early HTLV-1 infection events. Previous work demonstrated that mutation of the SRF and ELK-1 binding sites within the enhancer results in decreased viral gene expression *in vitro* (19). The results from our study showed that transient transfection of HTLV-1.mEnhancer proviral DNA into both HEK293T and Jurkat T-cells produced similar levels of sense and antisense viral transcripts and p19 Gag in the supernatant. In stable 729 HTLV-1.mEnhancer producer cells, p19 Gag levels in the supernatant as well as *tax* and *hbz* mRNA and protein levels were significantly decreased compared to those measured in 729 HTLV-1.wt cells. Although this could partially be attributed to location differences of intact viral genomes within the cellular genome (more or less transcriptionally active regions), one major difference is the short-term expression from transient transfections in HEK293T and Jurkat cells compared to the long-term cumulative effects from an intact viral genome in 729 cells.

Inoculation of NZW rabbits with 729 HTLV-1.wt or 729 HTLV-1.mEnhancer producers led to the establishment of persistent infection, marked by increases in proviral load and HTLV-1-specific antibody response over time in animals from either condition. While viral gene expression was variable in individual rabbits and the mean copy numbers of Gag/pol and Hbz fluctuated between time points, our results are characteristic of working with outbred animals and consistent with previous studies (18, 24, 37). At week 12 post-infection, there was a significant decrease in both *gag/pol* and *hbz* mRNA expression in rPBMCs infected with HTLV-1.mEnhancer compared to HTLV-1.wt. Inoculating HIS mice with either wt or mEnhancer virus led to the development of lymphoproliferative disease induced by HTLV-1 infection. There were no significant differences in proviral load or *tax* and *hbz* mRNA levels between the HTLV-1.wt and HTLV-1.mEnhancer groups. Given that the mice recapitulate disease onset and rapid progression within a compact time frame following initial infection (5 weeks), this may not be the appropriate model to study the long-term effects of the enhancer element on viral gene expression and persistence. While there are limitations to both rabbit and HIS mouse animal models utilized in this study, each allows for the evaluation of numerous aspects of HTLV-1 biology, including transmission, viral replication, persistence, and the immune response in rabbits as well as HTLV-1-mediated disease development and pathology in HIS mice. Although we have previously examined viral replication kinetics in infected rabbits in a 12-week timeframe, we extended the current study to 25 weeks to monitor the increasing trends in proviral load and HTLV-1-specific antibody response that were observed in both the HTLV-1.wt and HTLV-1.mEnhancer conditions. Our data showed that each of these features of persistent HTLV-1 infection reached their highest levels by the endpoint and confirmed that HTLV-1.mEnhancer behaves similarly to the wt virus in a long-term *in vivo* study. Although we detected a decrease in *gag/pol* and *hbz* transcripts in rabbits at week 12, we failed to detect a difference between the different animal groups at later time points. This suggests that while the viral enhancer may contribute to Hbz expression, there are alternative factors (both from the virus and the host) which regulate viral mRNA levels within host cells.

Our data from long-term *in vitro* immortalization assays showed that mutation of the viral enhancer did not hinder the ability of HTLV-1 to immortalize T-cells, since both HTLV-1.wt- and HTLV-1.mEnhancer-infected primary human lymphocytes showed similar efficiencies of immortalization. Both conditions had accumulation of p19 Gag in the culture supernatant and sufficiently immortalized target T-cells. Somewhat surprisingly, the cells immortalized by HTLV-1.mEnhancer had an altered immortalization phenotype and were predominantly CD8^+^ T-cells. Although HTLV-1 can infect both CD4^+^ and CD8^+^ T-cells, immortalized cells *in vitro*, infected cells in patients with HTLV-1, and patients with HTLV-1-mediated disease predominantly carry infected CD4^+^ T-cells (43). Previous studies have found that this preferential transformation tropism is dictated by the Env gene, not at the level of entry but after infection (24, 53). We found that while HTLV-1.mEnhancer immortalized cells did produce *tax, hbz*, and *env* transcript, the abundance of these transcripts was 2-3 log orders of magnitude lower than HTLV-1.wt immortalized cells. Expression of other HTLV-1 regulatory genes (p12, p30, and p13) was also lower in immortalized cell lines in the enhancer mutant condition compared to wt. Interestingly, PBLs which lacked the viral enhancer element consistently had 1-4 proviral integrations per cell compared to 10-40-fold more integrations in HTLV-1.wt immortalized cells. In the co-culture immortalization assay, the HTLV-1.mEnhancer infected cells produced lower levels of p19 Gag compared to HTLV-1.wt infected cells, and the wild-type cell lines had far more integrated proviruses. This is likely due to re-infection of the wild-type PBL cell lines, which produce sufficient levels of viral transcripts compared to the enhancer mutant PBL cell lines that had decreased levels of viral transcription. Although it would appear that enhanced viral transcription is favorable for immortalized cells *in vitro*, the HTLV-1.wt immortalized PBLs had higher levels of apoptosis than the HTLV-1.mEnhancer immortalized PBLs. Tax has been shown to induce rapid cell senescence driven by the transcriptional activity of NF-κB (54), therefore it is reasonable that immortalized cells with more Tax expression might have higher levels of apoptosis than those with lower Tax expression. Previous studies found that the ability of Tax to activate the HTLV-1 LTR is enhanced in CD4^+^ T-cells compared to CD8^+^ T-cells. This difference could account for the higher frequency of CD4^+^ HTLV-1-immortalized cells compared to CD8^+^ HTLV-1-immortalized cells (55). Subsequent studies found that Tax and viral LTR do not confer distinct transformation tropism (53). It is possible that the level of viral transcripts in an infected cell is critical to establish infection and influences the immortalization phenotype. Future studies should aim to determine whether the difference in the phenotype of cells immortalized by HTLV-1.mEnhancer is recapitulated *in vivo*. It would be informative to analyze live PBMC populations isolated from the animal models for the predominance of CD3^+^CD4^+^ or CD3^+^CD8^+^ T-cells. In addition, it would be useful to measure proviral load and viral gene expression in purified CD4^+^ or CD8^+^ T-cells from rabbits and HIS mice infected with HTLV-1.wt or HTLV-1.mEnhancer virus.

We were surprised by the result that cells immortalized by HTLV-1.mEnhancer had a higher percentage of cells in G0/G1 of the cell cycle, but lower levels of apoptosis compared to cells infected with HTLV-1.wt. Other studies have shown that Tax can trigger cell cycle arrest in the G1 phase in HeLa and human osteosarcoma (HOS) cells, as well as the human T lymphoblastic cell line SupT1 (56, 57). However, cell cycle arrest has also been recently identified in CD4+ T-cells from HTLV-1 asymptomatic carriers (58). Although the mechanisms underlying this observation are beyond the scope of this study, delayed cell division is in line with the slower proliferation rate of cells infected with HTLV-1.mEnhancer and may contribute to viral persistence.

A relationship between SRF/ELK-1 and HTLV-1, outside the context of the viral enhancer, has been demonstrated by previous studies (59). SRF and ELK-1 bind within the Tax-responsive element 2 of the U3 region of the 5’ LTR (60). Tax-mediated activation of SRF may play a key role in HTLV-1 pathogenesis, as this transcription factor induces expression of many genes involved in the regulation of cell growth, including c-fos, c-Jun, JunD, Erg-1, Erg-2, and Fra-1 (61–63). Dysregulation of SRF may be advantageous during multiple phases of HTLV-1 infection, such as the immortalization/transformation process as well as survival of leukemic cells. Defective proviruses are often found in ATL cells, with preferential selection for sequences where the 5’ LTR is deleted (64–66); thus, the maintenance of the vCTCF-BS and viral enhancer near the 3’ LTR provides other avenues for HTLV-1 to manipulate cellular signaling in host gene expression. Indeed, analysis of PBMCs from ATL patients showed readthrough transcription in host genes that flanked a provirus lacking the 5’ LTR (19).

Our results demonstrate that the viral enhancer element can influence viral gene transcription *in vivo*, but loss of this element does not affect establishment of persistent infection in rabbits or the induction of lymphoproliferative disease in HIS mice. Although abrogation of the viral enhancer does not affect the capacity of HTLV-1 to immortalize primary T-cell in culture, it does significantly alter the immortalization phenotype and viral gene expression. Other *in vitro* work has demonstrated that enhancer mutations abrogate the binding of SRF/ELK-1 and reduce host transcription near viral integration sites in HTLV-1-infected cells (19). This may indicate that the viral enhancer plays a larger role in disease progression of patients with ATL or pathogenic outcomes in individuals infected with HTLV-1. Future studies should address whether the viral enhancer cooperates with other regulatory elements, such as the vCTCF-BS, to promote transcription of viral genes and elicit changes in activity or organization of the surrounding host chromatin. Other studies should be performed to discern whether this enhancer element alters host gene transcription during early infection and disease development or alters the infected cell phenotype *in vivo.*

## Data availability statement

The raw data supporting the conclusions of this article will be made available by the authors, without undue reservation.

## Ethics statement

The studies involving human participants were reviewed and approved by The Ohio State University Institutional Review Board. The patients/participants provided their written informed consent to participate in this study. The animal studies were reviewed and approved by The Ohio State University Institutional Animal Care and Use Committee.

## Author contributions

AP and PG conceived and designed the study. VM and AP performed the experiments with support from SS and JS. CP performed the experiments in humanized mice with supervision from SN. VM, AP, and PG analyzed the data. YS provided the enhancer sequence and contributed to data analysis. VM and AP wrote the manuscript. All authors critically reviewed and approved the manuscript.

## Funding

This work was supported by National Cancer Institute, P01CA100730, and The Ohio State University Pelotonia Fellowship Program.

## Acknowledgments

We thank The Ohio State University Laboratory Animal Resources for their technical expertise in animal protocol design and the Genomics Shared Resource at The Ohio State University Comprehensive Cancer Center for Sanger sequencing services (CCSG: P30CA016058). We are grateful to Dr. Lianbo Yu from the Department of Biomedical Informatics at The Ohio State University for aid in the statistical analyses for our in vivo studies. We also thank members of the Panfil and Green laboratories for valuable discussions.

## Conflict of interest

The authors declare that the research was conducted in the absence of any commercial or financial relationships that could be construed as a potential conflict of interest.

## Publisher’s note

All claims expressed in this article are solely those of the authors and do not necessarily represent those of their affiliated organizations, or those of the publisher, the editors and the reviewers. Any product that may be evaluated in this article, or claim that may be made by its manufacturer, is not guaranteed or endorsed by the publisher.
